# Depression and non-suicidal self-injury: the mediating roles of childhood trauma and impulsivity

**DOI:** 10.3389/fpsyt.2025.1580235

**Published:** 2025-06-20

**Authors:** Wenjing Su, Hao Liu, Xiaoyan Zhou, Xueping Huang

**Affiliations:** ^1^ Center for Sleep Medicine, Chongqing Mental Health Center, Chongqing, China; ^2^ Department of Clinical Psychology, Chongqing Mental Health Center, Chongqing, China

**Keywords:** non-suicidal self self-injury, depression, childhood trauma, impulsivity, contributing factors

## Abstract

**Background:**

This study investigates the factors contributing to non-suicidal self-injury (NSSI) in individuals with depressive disorders, with a focus on how childhood trauma and impulsivity may function as mediating mechanisms. The findings aim to establish a theoretical framework that enhances clinicians’ ability to assess suicide-related risks in this population more accurately and promptly.

**Methods:**

This cross-sectional study enrolled patients diagnosed with depression who were hospitalized at Chongqing Mental Health Center between June 2019 and November 2021. All participants completed self- report questionnaires assessing demographic characteristics, NSSI behaviors, depression, anxiety, childhood trauma, impulsivity, family environment, parenting, and experiences in close relationships. Participants were divided into two groups based on NSSI behavior: the NSSI group and the non-NSSI group.

**Results:**

A total of 265 patients were included, 26.79% of whom were male. The NSSI group consisted of 150 patients (mean age = 17.50 ± 3.88 years), while the non-NSSI group included 115 patients (mean age = 23.06 ± 6.92 years). Binary logistic regression analysis identified several factors significantly associated with NSSI in patients with depression: gender (OR = 2.254, 95% CI: 1.083–4.693, *P* = 0.03), age (OR = 0.776, 95% CI: 0.708–0.851, *P* < 0.001), number of hospitalizations (OR = 1.747, 95% CI: 1.128–2.705, *P* = 0.012), suicide attempts (OR = 14.131, 95% CI: 4.023–49.64, *P* < 0.001), relationship duration (OR = 1.031, 95% CI: 1.009–1.045, *P* = 0.005), anxiety (OR = 1.03, 95% CI: 1.006–1.054, *P* = 0.015), sexual abuse (OR = 1.158, 95% CI: 1.036–1.294, *P* = 0.01), and negative thoughts (OR = 3.108, 95% CI: 1.101–8.774, *P* = 0.032). In addition, childhood trauma and impulsivity were found to partially mediate the relationship between depressive symptoms and NSSI, accounting for a total indirect effect of 20.52%.

**Conclusions:**

This study identifies multiple factors contributing to NSSI among individuals with depression. Notably, childhood trauma and impulsivity partially mediate the relationship between depressive symptoms and NSSI. These findings offer valuable insights for developing targeted prevention and intervention strategies to address NSSI in this population.

## Background

Depression is a complex group of mood disorders characterized by significant and persistent emotional disturbances, often accompanied by varying degrees of cognitive and behavioral dysfunction (1). As a multifactorial condition, depression affected more than 300 million individuals worldwide as of 2015 (2, 3). It is also a well-established risk factor for non-suicidal self-injury (NSSI), particularly among adolescents and young adults (4). NSSI refers to intentional self-inflicted harm without suicidal intent, typically manifested through behaviors such as cutting, burning, stabbing, hitting, or excessive rubbing acts generally considered socially unacceptable (5). These behaviors are not confined to psychiatric populations and may also be observed in the general community. The fifth edition of the *Diagnostic and Statistical Manual of Mental Disorders* (DSM-5) recognizes NSSI as a condition warranting further clinical study and proposes diagnostic criteria for its assessment (6). NSSI typically emerges during adolescence, with high prevalence rates reported globally. In China, the detection rate is approximately 27.4 % and has shown a steady upward trend in recent years (7, 8). While the incidence of NSSI tends to decline in adulthood, such behaviors may persist into young and middle adulthood, and even into later life (5, 9). This study includes participants aged 12 to 45 years, encompassing both adolescent and adult patients, which allows for comparative analysis of NSSI risk factors across age groups and enhances the comprehensiveness of the investigation.

Beyond its immediate physical and psychological consequences, NSSI is also considered a potential precursor to future suicidal behavior (9). Some researchers have suggested that NSSI and suicide may lie on a behavioral continuum, with repetitive self-injury potentially progressing toward suicidal actions (10). As the frequency and variety of NSSI behaviors increase, individuals may experience reduced pain sensitivity and diminished fear of death, thereby heightening the risk of engaging in more severe self-harm or suicide attempts (11). Longitudinal studies have shown that the suicide rate within one year among individuals with a history of NSSI is approximately 4.39‰, representing a 37- to 46-fold higher risk compared to the general population. Those who engage in violent forms of self-injury face the highest suicide risk within the first month following their initial act of self-harm (12, 13). Without timely and effective intervention, NSSI can lead to serious, life-threatening consequences.

A history of NSSI is one of the strongest predictors of future suicidal ideation and behavior, emphasizing the critical need for early identification of relevant risk factors (14). Research has consistently shown that the severity of depressive symptoms is positively associated with the occurrence of NSSI (15), while childhood trauma not only serves as an independent risk factor but also significantly predicts the onset of such behaviors (15, 16). Exposure to any type of childhood trauma increases the likelihood of NSSI by 2.–7 to 6.1 times (17). Additionally, individuals who engage in NSSI often exhibit high levels of impulsivity, which are linked to more frequent and severe forms of self- harm (18, 19). However, despite growing interest in the field, there remains a lack of in-depth exploration into the mechanisms underlying the association between depression and NSSI, particularly regarding family and school-related environmental influences.

Given the multitude of factors influencing NSSI, this study aims to identify key risk factors and behavioral patterns beyond those explored in single-mechanism models. By incorporating childhood trauma and impulsivity as mediating variables, this study seeks to clarify how these factors shape the relationship between depressive symptoms and NSSI. It also aims to examine both the mediating and moderating effects of childhood trauma and impulsivity within this framework. If these hypotheses are supported, the findings may inform effective intervention strategies to reduce the occurrence and progression of NSSI, thereby offering valuable references for clinical prevention and treatment.

Accordingly, the study proposes the following hypotheses regarding NSSI behavior:

1. Childhood trauma mediates the relationship between depressive symptoms and NSSI behavior.
2. Impulsivity mediates the relationship between depressive symptoms and NSSI behavior.
3. Childhood trauma and impulsivity simultaneously mediate the relationship between depressive symptoms and NSSI behavior in parallel.

## Methods

### Study design and participants

This cross-sectional study was conducted among patients diagnosed with depression who were hospitalized in the Clinical Psychology Department of Chongqing Mental Health Center between June 2019 and November 2021. Ethical approval was obtained from the Ethics Committee of Chongqing Mental Health Center. All participants met the inclusion criteria and provided informed consent. Adult participants completed the questionnaires independently, whereas minors completed them under the supervision of a guardian.

Inclusion criteria (1): A diagnosis of depressive disorder according to the DSM-5 criteria, regardless of prior use of antidepressants, including selective serotonin reuptake inhibitors (SSRIs), serotonin-norepinephrine reuptake inhibitors (SNRIs), noradrenergic and specific serotonergic antidepressants (NaSSAs), tricyclic and tetracyclic antidepressants (TCAs), or monoamine oxidase inhibitors (MAOIs), with diagnosis confirmed by a physician holding the title of attending physician or above (2); Age between 12 and 45 years (3); Ability to communicate effectively and complete the questionnaire independently or with assistance (4); Voluntary participation by both patients and their families, with signed informed consent. Exclusion criteria (1): Severe physical illness or organic brain disease; (2) Comorbid psychiatric disorders or depression secondary to other medical conditions; (3) Determination by the research team that the participant exhibited inadequate compliance or was unable to complete the study procedures. Participants were categorized into two groups based on their history of non-suicidal self-injury (NSSI): the NSSI group (with a history of NSSI) and the non-NSSI group (with no such history).

### Measures

#### Self-developed general information questionnaire

This questionnaire collected demographic and clinical information, including gender, ethnicity, age, education level, occupation, only-child status, family structure, place of residence, family economic status, housing conditions, age at depression onset, onset characteristics, first episode status, frequency of non-suicidal self-injury (NSSI) behaviors, suicide risk, and social functioning. Suicide risk was categorized into three levels: (1) no suicidal ideation or behavior, (2) suicidal ideation only, and (3) suicide attempts. Social functioning was classified into three categories: (1) normal work/study performance, (2) decreased work/study ability, and (3) inability to work or study.

#### Chinese version of the Beck depression inventory-II

This 21-item scale assesses the severity of depressive symptoms over the past two weeks. Each item is rated on a 4-point scale (0– –3), yielding a total score ranging from 0 to 63, with higher scores indicating more severe depressive symptoms. The severity is categorized as follows: 0–13 indicates no depression, 14–19 mild depression, 20–28 moderate depression, and 29–63 severe depression (20). In the present study, the scale demonstrated excellent internal consistency, with a Cronbach’s α of 0.943.

#### Chinese version of the Beck anxiety inventory

This 21-item scale measures the severity of anxiety symptoms experienced over the past week. Each item is rated on a 4-point scale (1 –4), with total raw scores ranging from 21 to 84. Scores are converted to standardized values using the formula Y = int(1.19x) (21). A score of ≥45 indicates the presence of anxiety symptoms, with higher scores reflecting greater severity. In this study, the Cronbach’s α for the scale was also 0.943, indicating high reliability.

#### Childhood trauma questionnaire

The CTQ assesses traumatic experiences during childhood across five domains: emotional abuse, physical abuse, sexual abuse, emotional neglect, and physical neglect. It comprises 28 items rated on a 5-point Likert scale (1–5) (22). Moderate to severe childhood trauma is defined as follows: emotional abuse ≥13, emotional neglect ≥15, sexual abuse ≥8, physical abuse ≥10, and physical neglect ≥10. Participants whose scores in all five domains fall below these thresholds are classified as having no significant childhood trauma. In the present study, the Cronbach’s α for this scale was 0.790.

#### Chinese version of the Barratt impulsiveness scale version 11

The BIS-11 is a 30-item instrument used to assess impulsivity, comprising three subscales: non-planning impulsivity, motor impulsivity, and attentional (cognitive) impulsivity. Each item is rated on a 5-point Likert scale (1 –5), with higher scores indicating greater impulsivity (23). In this study, the Cronbach’s α was 0.732.

#### Family environment scale Chinese version

The FES-CV consists of 90 items spanning 10 subscales that evaluate various aspects of the family’s social and environmental dynamics, including cohesion, expressiveness, conflict, independence, achievement orientation, intellectual–cultural orientation, active–recreational orientation, moral–religious emphasis, organization, and control. The third revised edition by Fei Lipeng was used in this study (24). The Cronbach’s α for this scale was 0.829.

#### Egma minnen av barndoms uppfostran

The EMBU measures perceived parenting styles, with 58 items assessing paternal behaviors and 57 items assessing maternal behaviors. It evaluates key parenting dimensions including emotional warmth, severity of punishment, rejection, favoritism, over-involvement, and over-protection (25). Items are rated on a 4-point scale (1–4), with some items reverse scored. The Cronbach’s α for the scale in this study was 0.941, indicating excellent internal consistency.

#### Experience in close relationships inventory revised

The ECR-R consists of 36 items rated on a 7-point Likert scale (1–7), with 13 items reverse scored. It assesses two core dimensions of adult attachment: attachment anxiety and attachment avoidance (26). Mean scores are used to classify individuals into four attachment styles: secure, preoccupied, dismissing, and fearful. The latter three are collectively referred to as insecure attachment. The Cronbach’s α for the scale in this study was 0.841.

### Statistical analyses

All statistical analyses were performed using SPSS version 26.0 (IBM Corp., Armonk, NY, USA). Continuous variables were presented as mean ± standard deviation (x̅ ± s), and all data conformed to a normal distribution. Independent samples t-tests were used for group comparisons. Categorical variables were reported as frequencies and percentages (%) and compared using the chi-square (χ²) test. Spearman correlation analysis was used to examine associations between variables. Factors influencing NSSI were identified through binary logistic regression. Mediation analyses were conducted using the PROCESS macro (Model 4) in SPSS, with bootstrap resampling ( 5,000 iterations) to estimate 95% confidence intervals (CIs) for indirect effects. A two-tailed P-value < 0.05 was considered statistically significant.

## Results

### Group differences in demographic and clinical characteristics

A total of 265 patients were included in the study, of whom 26.79% were male. Among them, 150 were assigned to the NSSI group (mean age: 17.50 ± 3.88 years) and 115 to the non-NSSI group (mean age: 23.06 ± 6.92 years). Significant differences were found between the two groups in terms of age, age group, gender, education level, occupation, monthly family income, age at onset, first episode status, number of relapses, number of hospitalizations, suicide risk, and duration of romantic relationships (all P < 0.05) (**Table 1**). Within the NSSI group, the frequency of NSSI behaviors was reported as follows: fewer than 3 instances in 28 cases (19.18%), 3–10 instances in 41 cases (28.08%), and more than 10 instances in 77 cases (52.74%).

**Table 1:** Comparison of demographic and clinical characteristics between the NSSI group and the non-NSSI group.

	NSSI group (n=150)	Non-NSSI group (n=115)	P
Age (years)	17.50 ± 3.88	23.06 ± 6.92	<0.001
Age group			<0.001
Adolescent (≤18 years)	106 (70.67)	30 (26.09)	
Adult (>18 years)	44 (29.33)	85 (73.91)	
Gender			<0.001
Male	25 (16.67)	41 (35.67)	
Female	125 (83.33)	74 (64.33)	
Ethnicity			0.474
Han	137 (91.33)	102 (88.70)	
Ethnic minorities	13 (8.66)	13 (11.30)	
Education			<0.001
Primary and below	1 (0.67)	0 (0)	
Middle school	47 (31.33)	12 (10.43)	
High school	63 (42.00)	33 (28.70)	
College and above	39 (26.00)	70 (60.87)	
Occupation			<0.001
Student	126 (84.00)	61 (53.04)	
Official	0 (0)	5 (4.35)	
Worker	1 (0.67)	1 (0.87)	
Unemployed	10 (6.67)	19 (16.52)	
Individual	0 (0)	7 (6.09)	
Employee	13 (8.67)	77 (19.13)	
Only child			0.129
No	96 (64.00)	63 (54.78)	
Yes	54 (36.00)	52 (45.22)	
Family Composition			0.292
Original family	117 (78.00)	84 (73.04)	
Divorced family	1 (12.67)	13 (11.30)	
Reconstructed family	14 (9.33)	18 (15.66)	
Residence			0.125
Urban	126 (84.00)	104 (90.43)	
Rural	2 (16.00)	11 (9.57)	
Monthly Income (RMB)			0.019
<2000	12 (8.00)	4 (3.48)	
2000-5000	58 (38.67)	27 (23.48)	
5001-8000	30 (20.00)	35 (30.43)	
8001-10000	24 (16.0)	20 (17.39)	
>10000	26 (17.33)	29 (25.22)	
Housing Conditions			0.981
<60m^2^	22 (14.67)	16 (13.91)	
60-100m^2^	82 (54.67)	64 (55.65)	
>100m^2^	46 (30.66)	35 (30.44)	
Onset of Illness			0.375
Acute	28 (18.67)	21 (18.26)	
Subacute	18 (12.00)	8 (6.96)	
Chronic	104 (69.33)	86 (74.78)	
Onset Age (years)	14.43 ± 3.54	19.39 ± 6.26	<0.001
Initial Diagnosis			0.015
First Episode	74 (49.33)	74 (64.35)	
Relapse	76 (50.67)	41 (35.65)	
Number of Relapses	4.45 ± 10.03	2.17 ± 2.64	0.008
Number of Hospitalizations	1.00 ± 0.67	0.54 ± 0.92	<0.001
Suicide Risk			<0.001
None suicide risk	11 (7.33)	24 (20.87)	
Negative Thoughts	73 (48.67)	83 (72.17)	
Suicide Attempts	66 (44.00)	8 (6.96)	
Social Function			0.264
Normal Work/Study	17 (11.33)	12 (10.44)	
Decreased Work/Study Ability	98 (65.33)	85 (73.91)	
Unable to Work/Study	35 (23.33)	18 (15.65)	
Current Relationship Status			0.657
In a Relationship	47 (31.33)	39 (33.91)	
Not in a Relationship	103 (68.67)	76 (66.09)	
Previous Relation Status			0.947
Yes	88 (58.67)	67 (58.26)	
No	62 (41.33)	48 (41.74)	
Duration of Relationship (months)	9.70 ± 15.84	14.33 ± 20.98	0.049

### Comparison of BDI-II-C, BAI-C, CTQ, BIS-11, FES-CV, EMBU, and ECR-R scores between groups

The detection rate of childhood trauma was 91.33% (137/150) in the NSSI group and 92.17% (106/115) in the non-NSSI group, with no statistically significant difference (P = 0.806). According to the ECR- R, attachment styles in the NSSI group were distributed as follows: secure (2.67%, n = 4), fearful (65.33%, n = 98), preoccupied (22.67%, n = 34), and dismissive (9.33%, n = 14), with 97.33% ( n = 146) classified as having insecure attachment. In the non-NSSI group, the distribution was: secure (9.57%, n =11), fearful (66.08%, n = 76), preoccupied (19.13%, n = 22), and dismissive (5.22%, n = 6), with 90.43% ( n = 104) classified as having insecure attachment. While there was no significant difference in the distribution of the four attachment styles between groups (P = 0.062), the difference in secure versus insecure attachment was statistically significant (P = 0.016).

Significant differences were observed between the two groups in BDI-II-C scores (31. ± 20 ± 11.57 vs. 26. ± 01 ± 10.12, P = 0.002) and BAI-C scores (55. ± 35 ± 14.87 vs. 48. ± 46 ± 14.16, P < 0.001). The total CTQ score was significantly higher in the NSSI group (57. ± 57 ± 13.16 vs. 49. ± 57 ± 7.27, P < 0.001), with notable differences in emotional abuse (13. ± 17 ± 4.76 vs. 9. ± 74 ± 3.67, P < 0.001), physical abuse (10. ± 41 ± 5.15 vs. 7. ± 12 ± 2.94, P < 0.001), sexual abuse (8. ± 45 ± 4.68 vs. 6. ± 37 ± 2.28, P < 0.001), and emotional neglect (13. ± 81 ± 4.75 vs. 15. ± 10 ± 5.05, P = 0.034).

In terms of impulsivity (BIS-11), the NSSI group had higher total scores (93. ± 11 ± 20.06 vs. 87. ± 77 ± 15.59, P = 0.015), as well as higher scores on the non-planning (33. ± 84 ± 8.39 vs. 31. ± 82 ± 7.14, P = 0.039), behavioral (30. ± 35 ± 7.20 vs. 27. ± 52 ± 6.10, P = 0.001), and cognitive (30. ± 22 ± 6.99 vs. 28. ± 43 ± 6.28, P = 0.029) impulsivity subscales.

Within the family environment (FES-CV), significant differences were observed in the dimensions of ± cohesion (4.30 ± 2.33 vs. 4. ± 97 ± 2.57, P = 0.027), conflict (5. ± 43 ± 2.36 vs. 4. ± 51 ± 2.37, P = 0.002), and ± expressiveness (3.03 ± 2.10 vs. 2. ± 50 ± 1.74, P = 0.032).

For parenting styles (EMBU), significant group differences were found in paternal emotional warmth and understanding (39. ± 28 ± 11.98 vs. 43. ± 37 ± 11.49, P = 0.005), punishment severity (22. ± 79 ± 9.11 vs. 20. ± 23 ± 6.37, P = 0.008), favoritism (8. ± 97 ± 3.82 vs. 10. ± 13 ± 3.90, P = 0.016), and rejection and denial (12. ± 35 ± 4.69 vs. 11. ± 18 ± 3.86, P = 0.027) (**Table 2**).

**Table 2:** Comparison of scores on BDI-II-C, BAI-C, CTQ, BIS-11, FES-CV, EMBU, and ECR-R between the NSSI and non-NSSI groups.

	NSSI group	Non-NSSI group	P
BDI-II-C	31.20 ± 11.57	26.01 ± 10.12	0.002
BAI-C	55.35 ± 14.87	48.46 ± 14.16	<0.001
CTQ			
Total score	57.57 ± 13.16	49.57 ± 7.27	<0.001
Emotional Abuse	13.17 ± 4.76	9.74 ± 3.67	<0.001
Physical Abuse	10.41 ± 5.15	7.12 ± 2.94	<0.001
Sexual Abuse	8.45 ± 4.68	6.37 ± 2.28	<0.001
Emotional Neglect	13.81 ± 4.75	15.10 ± 5.05	0.034
Physical Neglect	11.73 ± 3.26	11.24 ± 2.37	0.182
BIS-11			
Total Score	93.11 ± 20.06	87.77 ± 15.59	0.015
Planned Impulsivity	33.84 ± 8.39	31.82 ± 7.14	0.039
Action Impulsivity	30.35 ± 7.20	27.52 ± 6.10	0.001
Cognitive Impulsivity	30.22 ± 6.99	28.43 ± 6.28	0.029
FES-CV			
Intimacy	4.30 ± 2.33	4.97 ± 2.57	0.027
Conflict	5.43 ± 2.36	4.51 ± 2.37	0.002
Knowledge	3.03 ± 2.10	2.50 ± 1.74	0.032
Emotional expression	3.35 ± 1.63	3.59 ± 1.87	0.258
Independence	5.35 ± 1.76	5.35 ± 1.60	0.996
Success	5.35 ± 2.03	5.16 ± 2.08	0.440
Entertainment	3.21 ± 2.08	3.44 ± 2.44	0.419
Moral and Religious View	4.39 ± 1.65	4.32 ± 1.60	0.723
Organization	4.64 ± 2.09	4.97 ± 2.37	0.238
Control	3.47 ± 2.31	3.12 ± 2.21	0.220
EMBU			
Father's Emotional Warmth, Understanding	39.28 ± 11.98	43.37 ± 11.49	0.005
Father's Punishment, Severity	22.79 ± 9.1155	20.23 ± 6.367	0.008
Father's Over-Interference	21.95 ± 6.97	20.94 ± 5.92	0.205
Father's Favoritism	8.97 ± 3.82	10.13 ± 3.90	0.016
Father's Rejection, Denial	12.35 ± 4.69	11.18 ± 3.86	0.027
Father's Over-Protectiveness	13.30 ± 4.60	12.98 ± 3.49	0.523
Mother's Emotional Warmth, Understanding	41.92 ± 11.68	45.52 ± 12.02	0.015
Mother's Over-Interference, Over-Protectiveness	40.03 ± 11.70	36.24 ± 10.04	0.005
Mother's Rejection, Denial	17.78 ± 6.64	15.18 ± 5.30	<0.001
Mother's Punishment, Severity	17.77 ± 7.25	14.86 ± 5.30	<0.001
Mother's Favoritism	9.00 ± 3.72	10.13 ± 4.03	0.019
ECR-R			
Avoidant Attachment Mean	3.94 ± 1.19	3.62 ± 1.03	0.021
Anxious Attachment Mean	4.52 ± 0.95	4.11 ± 0.83	<0.001

### Correlation analysis

Emotional neglect (CTQ), family cohesion (FES-CV), and parental emotional warmth, understanding, and favoritism (EMBU) were negatively correlated with NSSI behavior among patients with depression (r = –0.170 to –0.130, P < 0.05). Conversely, the severity of depression and anxiety, the total CTQ score and subscales ( emotional, physical, and sexual abuse), the total BIS-11 score and subscales (non- planning, behavioral, and cognitive impulsivity), family conflict and control (FES-CV), paternal rejection and punishment, maternal over-involvement, over-protection, rejection, and punishment (EMBU), as well as attachment avoidance and anxiety (ECR- R), were all positively correlated with NSSI behavior (r = 0.127 to 0.367, P < 0.05) (**Table 3**).

**Table 3:** Correlation analysis of scores on various dimensions of scales with NSSI behavior in depressed patients.

Scale	NSSI behavior	
	R value	P
BDI-II-C	0.364	<0.001
BAI-C	0.229	<0.001
CTQ		
Total score	0.340	<0.001
Emotional Abuse	0.367	<0.001
Physical Abuse	0.354	<0.001
Sexual Abuse	0.262	<0.001
Emotional Neglect	-0.130	0.034
Physical Neglect	0.082	0.182
BIS-11		
Total Score	0.144	0.019
Planned Impulsivity	0.127	0.039
Action Impulsivity	0.204	0.001
Cognitive Impulsivity	0.132	0.031
FES-CV		
Intimacy	-0.136	0.027
Conflict	0.190	0.002
Knowledge	0.132	0.032
Emotional expression	-0.070	0.258
Independence	<0.001	0.996
Success	0.048	0.440
Entertainment	-0.051	0.409
Moral and Religious View	0.022	0.723
Organization	-0.073	0.238
Control	0.076	0.220
EMBU		
Father's Emotional Warmth, Understanding	-0.170	0.005
Father's Punishment, Severity	0.156	0.011
Father's Over-Interference	0.076	0.215
Father's Favoritism	-0.148	0.016
Father's Rejection, Denial	0.132	0.032
Father's Over-Protectiveness	0.038	0.538
Mother's Emotional Warmth, Understanding	-0.150	0.015
Mother's Over-Interference, Over-Protectiveness	0.169	0.006
Mother's Rejection, Denial	0.207	0.001
Mother's Punishment, Severity	0.218	<0.001
Mother's Favoritism	-0.144	0.019
ECR-R		
Avoidant Attachment Mean	0.141	0.021
Anxious Attachment Mean	0.220	<0.001

### Binary logistic regression analysis of factors affecting NSSI

Binary logistic regression analysis identified the following variables as significant predictors of NSSI behavior in patients with depression: gender (OR = 2.254, 95% CI : 1.083–4.693, P = 0.03), age (OR = 0.776, 95% CI : 0.708–0.851, P < 0.001), number of hospitalizations (OR = 1.747, 95% CI : 1.128–2.705, P = 0.012), history of suicide attempts (OR = 14.131, 95% CI : 4.023–49.640, P < 0.001), duration of romantic relationships (OR = 1.031, 95% CI : 1.009–1.045, P = 0.005), BAI-C score (OR = 1.030, 95% CI : 1.006–1.054, P = 0.015), experience of sexual abuse (OR = 1.158, 95% CI : 1.036–1.294, P = 0.01), and presence of negative thoughts (OR = 3.108, 95% CI : 1.101–8.774, P = 0.032) (**Table 4**).

**Table 4:** Binary logistic regression analysis of factors affecting NSSI.

	OR	95% CI		P
Gender	2.254	1.083	4.693	0.030
Age	0.776	0.708	0.851	<0.001
Number of Hospitalizations	1.747	1.128	2.705	0.012
Suicide Risk (None)				<0.001
Negative Thoughts	3.108	1.101	8.774	0.032
Suicide Attempt	14.131	4.023	49.640	<0.001
Relationship Duration	1.031	1.009	1.054	0.005
BAI-C	1.030	1.006	1.054	0.015
Childhood Sexual Abuse	1.158	1.036	1.294	0.010
Constant	3.456			0.321

### Mediating effects of childhood trauma and impulsivity in the relationship between depressive mood and NSSI behavior

**Figure 1:**
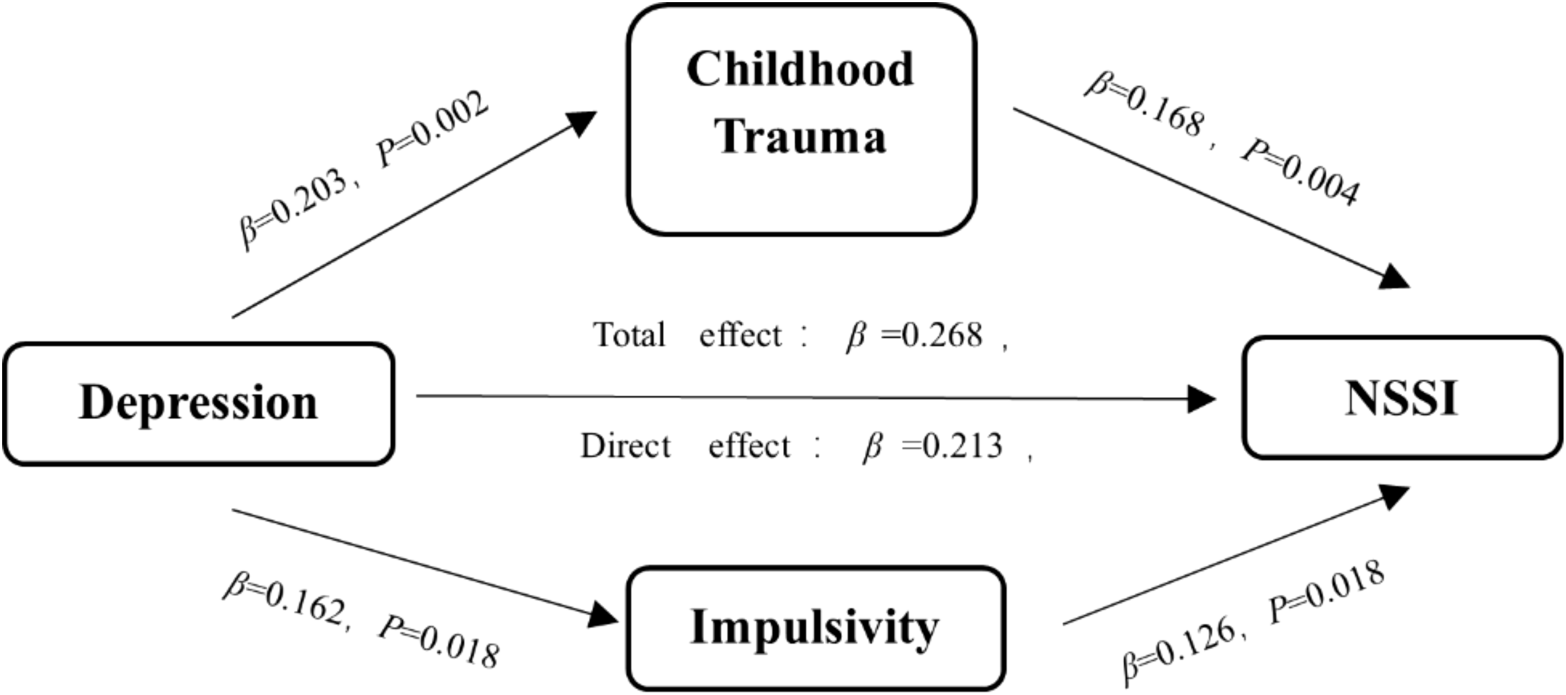
Mediation model illustrating the roles of childhood trauma and impulsivity in the relationship between depressive mood and NSSI behavior.

To investigate the mechanisms underlying the significant positive association between depressive mood and NSSI behavior, childhood trauma and impulsivity were introduced as parallel mediators. The BDI-II-C score served as the predictor variable, and the frequency of NSSI behavior as the outcome variable, as illustrated in **Figure 1**. Gender, age, education level, academic major, and family income were controlled for in the regression analysis. Results showed that depressive mood significantly predicted NSSI behavior (β = 0.268, *P* < 0.001). After incorporating childhood trauma and impulsivity into the model, the predictive effect of depressive mood on NSSI behavior decreased (β = 0.213, *P* < 0.001). Additionally, depressive mood significantly predicted both childhood trauma (β = 0.203, *P* = 0.002) and impulsivity (β = 0.162, *P* = 0.018). Childhood trauma (β = 0.168, *P* = 0.004) and impulsivity (β = 0.126, *P* = 0.018) were also significant predictors of NSSI behavior. The total effect of depressive mood on NSSI behavior, as well as the indirect effects of childhood trauma and impulsivity, were both significant, with Bootstrap 95% confidence intervals not including zero. There was no significant difference between the mediating effects of childhood trauma and impulsivity (comparative effect size: 0.014, 95% CI: –0.027 to 0.052). These findings suggest that both childhood trauma and impulsivity partially mediate the relationship between depressive mood and NSSI behavior (indirect effect = 0.055, 95% CI: 0.022–0.091, *P* < 0.001), accounting for 20.52% of the total effect (**Tables 5**, **6** **;** **Figure 1**).

**Table 5:** Regression analysis of variable relationships in the mediation model.

Predictor variable	Outcome variable	Overall fit index			Regression Coefficient significance		
		*R*	*R2*	*F*	*β*	*t*	P
Depression	NSSI behavior	0.561	0.314	14.674	0.213	3.570	<0.001
Depression	Childhood Trauma	0.411	0.169	8.743	0.203	3.201	0.002
Depression	Impulsivity	0.215	0.046	2.088	0.162	2.385	0.028
Childhood Trauma	NSSI behavior	–	–	–	0.168	2.945	0.004
Impulsivity	NSSI behavior	–	–	–	0.126	2.376	0.018

**Table 6:** Bootstrap test results for the mediation effect of childhood trauma and impulsivity.

Item	Effect value	Boot SE	95% Confidence interval		Effect size
			Lower limit	Upper limit	
Direct Effect	0.213	0.008	0.012	0.043	79.48%
Indirect Effect of Childhood Trauma	0.034	0.140	0.009	0.063	12.69%
Indirect Effect of Impulsivity	0.021	0.013	<0.001^a^	0.049	7.83%
Total Indirect Effect	0.055	0.018	0.022	0.091	20.52%
Total Effect	0.268	0.008	0.020	0.050	–
Comparison of Indirect Effects of Childhood Trauma and Impulsivity	0.014	0.020	-0.027	0.052	–

The 95% confidence interval lower limit for the indirect effect of impulsivity is <0.00164, which is greater than 0.

## Discussion

This study identified multiple factors that may contribute to NSSI in individuals with depression. Notably, childhood trauma and impulsivity were found to partially mediate the relationship between depressive mood and NSSI, highlighting the importance of addressing these factors in the clinical treatment and prevention of NSSI. Targeting childhood trauma and impulsivity may improve intervention strategies and enhance outcomes for this vulnerable population. The prevalence of NSSI among patients with depression in this study was 56.60% (150/265), with a notably higher incidence in adolescents at 77.94% (106/136). More severe depressive symptoms were associated with a greater likelihood of engaging in NSSI. Age emerged as an independent risk factor: with each additional year of age, the risk of NSSI decreased by approximately 22.4% (15, 19). This may be due to younger individuals being more emotionally unstable and susceptible to external influences (27, 28). Adolescents often encounter psychological stressors such as bullying, academic pressure, interpersonal conflict, poor self-regulation, and peer influence—all of which heighten the risk of NSSI (29). Therefore, implementing psychological health screenings in schools is crucial to suicide prevention among adolescents. Although less prevalent in adults, NSSI behavior still persists and warrants clinical attention. Univariate analysis revealed significant differences between the NSSI and non-NSSI groups in various demographic, clinical, and family-related factors. Correlation analyses showed that family intimacy, parental warmth, and parental favoritism were negatively correlated with NSSI behavior, while depressive and anxiety symptoms, childhood trauma, impulsivity, family conflict, and harsh parenting were positively correlated. Family environment and parenting styles are closely linked to individual psychological development (30). These findings suggest that a nurturing family atmosphere and warm, understanding parenting can help mitigate the occurrence of NSSI, consistent with previous studies (31). Moreover, our study supports the view that an insecure attachment style is a risk factor for NSSI. Individuals with insecure attachment tend to have lower self-esteem and reduced social confidence, along with heightened anxiety and avoidance behaviors, which may lead them to engage in NSSI as a coping mechanism to relieve emotional distress or negative cognitive states (32).

Further analysis revealed that patients with multiple hospitalizations were more likely to exhibit NSSI behavior compared to those hospitalized for the first time. Additionally, a longer duration of romantic relationships was associated with a higher likelihood of engaging in NSSI. Female patients were 2.254 times more likely to engage in NSSI than male patients, consistent with findings from most prior studies (19, 33). Gender has emerged as a high-risk factor for NSSI, potentially due to differing emotional coping strategies: males are more inclined to distract themselves from depressive emotions, whereas females are more likely to internalize distress, often manifesting through complaints or somatization as defense mechanisms (34). Although some studies have reported a higher incidence of NSSI in males than in females (35), it remains essential to consider gender differences when designing NSSI intervention strategies.

Suicide risk was also identified as an independent high-risk factor for NSSI.

Patients with negative thoughts were 3.1 times more likely to engage in NSSI, and those with a history of suicide attempts were 14.1 times more likely to do so, compared to those without suicide risk. These findings support the theory that NSSI may elevate the risk of suicidal ideation and attempts, aligning with the continuum model, which posits a progression from self-harm to suicide (12, 13). Given this continuum, early detection and intervention for NSSI are crucial to mitigate the risk of suicide. Anxiety was also found to be a significant contributor to NSSI behavior (36, 37). Each one-point increase in the BAI-C score raised the risk of NSSI by 1.030 times. The overwhelming distress and emotional discomfort caused by anxiety may drive patients to seek rapid, tangible relief through self-harming behaviors such as cutting, as a temporary means of emotional escape. Effective management of anxiety symptoms can reduce the risk of NSSI through psychological and behavioral interventions, such as emotion recognition, encouraging emotional expression, exercise, meditation, mindfulness, deep breathing, and progressive muscle relaxation. Pharmacological treatment with anxiolytics may also be warranted when necessary.

In this study, 91.7% of patients with depression had experienced childhood trauma, with no significant difference in incidence between those with and without NSSI. However, the severity of childhood trauma —particularly emotional, physical, and sexual abuse —was positively correlated with NSSI. Binary logistic regression identified sexual abuse as an independent risk factor, with each one-point increase in the CTQ sexual abuse subscale increasing the risk of NSSI by 1.158 times. Patients with NSSI also exhibited higher levels of impulsivity (38, 39). Total BIS-11 scores, as well as scores on the non-planning, motor, and cognitive impulsivity subscales, were significantly higher in the NSSI group compared to controls, and impulsivity was positively associated with the risk of NSSI.

Consistent with expectations, the mediation analysis confirmed that childhood trauma and impulsivity functioned as parallel partial mediators in the relationship between depressive symptoms and NSSI behavior. There was no significant difference between the two mediation pathways: depression → childhood trauma → NSSI and depression → impulsivity → NSSI. These findings highlight childhood trauma and impulsivity as significant risk factors for NSSI among patients with depression, with their severity being positively correlated with the occurrence of NSSI—consistent with previous research (19, 40, 41). This may be attributed to immature psychological development, unstable personality traits, and incomplete cognitive maturity in childhood (42), which increase susceptibility to extreme behavioral responses under intense psychological distress (43, 44).

Moreover, childhood trauma has been linked to structural changes in the brain, such as reduced gray matter volume, cortical thinning, and alterations in brain regions including the amygdala and insula (45, 46). Neurobiological abnormalities—such as disrupted N-acetylaspartate (NAA) metabolism in the right thalamus and dysfunction in white matter tracts like the uncinate fasciculus and cingulate gyrus —can impair emotional regulation and elevate impulsivity (47). Consequently, individuals with a history of trauma may exhibit emotional dysregulation and impulsive tendencies when confronted with stress, leading them to adopt NSSI as a maladaptive coping strategy for temporary emotional relief and psychological escape.

In summary, multiple factors contribute to NSSI behavior in patients with depression. The severity of depressive symptoms positively predicts NSSI behavior, while childhood trauma and impulsivity serve as parallel mediators in this relationship. These findings suggest that both distal (e.g., childhood trauma) and proximal (e.g., impulsivity) factors should be considered when formulating NSSI prevention strategies. Community-level screenings may help identify high-risk families affected by trauma, and parent-child attachment programs should be promoted. For patients already exhibiting depressive symptoms, cognitive behavioral interventions may assist in reducing impulsive decision-making. Early identification of the risk factors contributing to NSSI—coupled with targeted prevention, timely intervention, and appropriate treatment —is crucial to lowering the incidence of NSSI and preventing its progression to more severe outcomes.

The innovation of this study lies in its first construction and validation of a moderated mediation model examining the relationship between depression and non-suicidal self-injury (NSSI) among Chinese adolescents and adults . This model reveals the crucial roles of childhood trauma and impulsivity in this relationship. These findings not only enhance our understanding of the mechanisms through which depression influences NSSI but also provide a theoretical foundation and practical guidance for the prevention and intervention of NSSI behaviors. Moreover, the results underscore the critical importance of the family and school environments in shaping mental health outcomes, offering valuable implications for the development of relevant policies and intervention strategies. This study also elucidates the specific impact of childhood emotional abuse on adolescent NSSI behavior, offering new perspectives and directions for future mental health interventions.

Despite the valuable insights gained, this study has several limitations. First, the sample size is relatively small, with notable imbalances in group sizes and gender distribution. Furthermore, the study did not stratify the specific characteristics of NSSI, such as the diversity of methods, timing, frequency, and severity, which could provide a more nuanced understanding of the behavior. Although demographic variables such as age and gender were controlled for, other potential influences—such as environmental stressors, genetic susceptibility, and neurobiological factors—may independently or interactively affect the mediation pathways. These require further investigation, ideally through multi-omics data integration.

Second, as a cross-sectional study, it relies on participants' retrospective self-reports to collect historical exposure data, such as experiences of childhood trauma . This method may introduce recall bias. Moreover, the study design does not allow for establishing a clear temporal relationship between depressive symptoms and NSSI behaviors. The possibility of a bidirectional relationship (e.g., NSSI may temporarily alleviate depressive symptoms but worsen emotional states over time) cannot be excluded. Future longitudinal studies are needed to verify the direction and causality of this association.

Third, since this was a case-control study based on self-reported data, there is a risk of recall bias, potentially leading to overreporting or underreporting of past experiences. Fourth, the study sample was limited to patients hospitalized in specialized psychiatric institutions. As such, some psychological or behavioral indicators may be elevated compared to those in the general population, which may limit the generalizability of the findings—particularly to individuals with mild symptoms or those who remain undiagnosed. In addition, most patients may have received outpatient treatment prior to hospitalization, possibly improving their self- injury conditions and influencing the study outcomes.

Therefore, future research should aim to include larger, more diverse samples and conduct longitudinal studies on the risk and protective factors influencing NSSI . Studies involving newly diagnosed and untreated patients will be particularly valuable for generating more comprehensive and accurate findings. Integrating interdisciplinary data may improve the spatiotemporal precision of intervention targets, shifting the research focus from descriptive associations to precision prevention.

## Conclusions

NSSI in individuals with depression is influenced by multiple factors, with childhood trauma and impulsivity serving as parallel partial mediators in this relationship. These findings provide important guidance for developing more targeted prevention and intervention strategies to reduce NSSI and its associated adverse outcomes.

## Data Availability

The original contributions presented in the study are included in the article/supplementary material. Further inquiries can be directed to the corresponding authors.
